# Metabolomic, proteomic and lactylated proteomic analyses indicate lactate plays important roles in maintaining energy and C:N homeostasis in *Phaeodactylum tricornutum*

**DOI:** 10.1186/s13068-022-02152-8

**Published:** 2022-05-31

**Authors:** Aiyou Huang, Yuanxiang Li, Jiawen Duan, Shiyi Guo, Xiaoni Cai, Xiang Zhang, Hao Long, Wei Ren, Zhenyu Xie

**Affiliations:** 1 State Key Laboratory of Marine Resource Utilization in the South China Sea, Hainan University, Haikou, Hainan 570228 People’s Republic of China; 2 Laboratory of Development and Utilization of Marine Microbial Resource, Hainan University, Haikou, Hainan 570228 People’s Republic of China; 3 Key Laboratory of Tropical Hydrobiology and Biotechnology of Hainan Province, Haikou, Hainan 570228 People’s Republic of China; 4 College of Marine Sciences, Hainan University, Haikou, Hainan 570228 People’s Republic of China; 5 Department of Pharmacology and Regenerative Medicine, University of Illinois at Chicago, Chicago, IL USA

**Keywords:** *Phaeodactylum tricornutum*, C:N ratio, Lactate, Lactylation modification, High CO_2_ concentration, Lipid

## Abstract

**Background:**

*Phaeodactylum tricornutum* accumulates lipids while the growth also increases under high CO_2_, shedding light on its potential application in the reduction of CO_2_ emissions and at the same time acquiring biodiesel raw materials. However, the sensing and transducing of high C:N signals and the related response mechanism(s) remained unknown.

**Results:**

In this study, a multiple omics analysis was performed with *P. tricornutum* under low nitrogen (LN) and high CO_2_ (HC) conditions. The results indicated that 2-oxoglutarate was significantly increased under both LN and HC. Meanwhile, proteins involved in carbon concentration mechanism decreased, indicated that 2-oxoglutarate might regulate C:N balance through suppressing carbon fixation. Lactate, which acts in energy metabolism, signal transduction and ‘LactoylLys’ modification on proteins, was the most upregulated metabolite under both LN and HC conditions. Meanwhile, proteins involved in carbon, nitrogen and energy metabolisms were significantly regulated. Western blotting analysis suggested that non-histone L-lactylation modification was enhanced under LN and HC. Moreover, lactylated proteins were enriched in photosynthesis, central carbon metabolism, nitrogen metabolism, fatty acid synthesis and oxidative phosphorylation.

**Conclusion:**

It is suggested that lactate might play important roles in energy homeostatic maintenance and C:N balance regulation in *P. tricornutum* through protein lactylation modification.

**Supplementary Information:**

The online version contains supplementary material available at 10.1186/s13068-022-02152-8.

## Background

Diatoms are important phytoplankton which are responsible for approximately 20% of global primary productivity [1, 2] and thus play an important role in carbon fixation and the material cycle on earth [3, 4]. The great success of diatoms is mainly due to their rapid response to fluctuations in nitrate in the ocean. Diatoms possess a complete urea cycle, which might facilitate the reassimilation of nitrogen in catabolic N-containing components [5], making them response quickly to nitrogen-limited conditions. In contrast to carbon regulation in green algae and higher plants, which increase carbon stores under nitrogen depletion conditions, diatoms accelerate glycolysis and the TCA cycle to provide carbon skeletons for nitrogen redistribution [6]. This response of diatoms under nitrogen-limited conditions is a result of the superior efficiency of intracellular carbon store breakdown as a source of carbon for the reassimilation of nitrogen than for photosynthesis [6]. Because of the importance of diatoms and their unique characteristics, the response of diatoms to nitrogen depletion has received much attention in the past decade.

The pennate diatom *Phaeodactylum tricornutum* (*P. tricornutum*) is considered a model organism for the study of physiology, evolution and biochemistry in single-cell organism due to its clear genomic background [7], universal molecular toolbox [8] and stable transgene expression system [9, 10]. In the past decade, the physiological response [11], gene expression and metabolic pathway regulation mechanisms of *P. tricornutum* under nitrogen deficiency have been studied [12–15]. Under nitrogen deficiency, *P. tricornutum* accumulates lipids at the expense of depressed growth, photosynthesis, chlorophyll biosynthesis, the Calvin cycle and chrysolaminarin biosynthesis, and increased recycling of N compounds, such as amino acids, proteins, chlorophyll and nucleic acids; high turnover of cell components (e.g. soluble sugar, membrane lipids and phosphatidylcholines) that reallocates cellular nutrients to glycolysis and the TCA cycle, which are accelerated to provide carbon skeletons for redistribution of nitrogen, was all observed [12, 16, 17]. However, the mechanisms of sensing and transducing nitrogen deficiency signals and the related responses have not been elucidated.

In cyanobacteria, 2-oxoglutarate (2-OG), the metabolic intermediate of TCA cycle, played an important role in the C:N balance regulation by binding to the key regulators PII and NtcA, two receptors in the nitrogen starvation signalling pathways. Our previous study indicated that 2-OG content was upregulated 22-fold in *P. tricornutum* under nitrogen-deficient conditions [18]. As an important metabolite of central carbon metabolism, 2-OG provides a carbon skeleton for the assimilation of nitrogen through glutamate synthase, connecting intracellular carbon metabolism and nitrogen metabolism [19, 20]. Thus, the accumulation of 2-OG indicates a high carbon: nitrogen (C:N) ratio, and accumulated 2-OG emits a nitrogen starvation signal, playing an important role in the regulation of carbon and nitrogen metabolism in cyanobacteria [21]. When the C:N ratio is high, 2-OG suppresses carbon fixation by downregulating genes involved in the Calvin cycle and the carbon concentration mechanism (CCM), which leads to elevated CO_2_ concentrations near Rubisco to enhance CO_2_ fixation [20, 21]. The accumulation of 2-OG in *P. tricornutum* under nitrogen-deficient conditions, together with changes in carbon metabolism in response to nitrogen starvation in diatoms bears a greater resemblance to the response of cyanobacteria than to that of higher plants or green algae, triggering our interest in investigating the role of 2-OG in *P. tricornutum.*

If 2-OG indeed plays a role in C:N balance regulation in *P. tricornutum*, it might also act under other conditions which can result in a high intracellular C:N ratio. Besides nitrogen depletion, high inorganic carbon concentration, i.e. ocean acidification induced by increasing of atmospheric CO_2_, can also result in a high intracellular C:N ratio. In fact, our previous study suggests some similar response of *P. tricornutum* to high CO_2_ concentration as to low nitrogen, including accumulation of lipid, increase in glycolysis and TCA cycle activities and remodelling of cellular components [22, 23]. We propose that nitrogen deficiency or high CO_2_ concentration essentially disrupts the intracellular C:N balance and energy balance, thus triggering a series of signal transduction and regulation processes to maintain cell homeostasis. In contrast to cyanobacteria, *P. tricornutum* is an oleaginous microalga in which lipids accumulate under high C:N ratio conditions. This lipid accumulation is accompanied with remodelling of intracellular components and redistributing of metabolites. Hence, carbon and nitrogen level regulation may not be limited to the carbon and nitrogen assimilation; it may also be related to the degradation and redirection of intracellular catabolic products.

Although researchers have investigated the response to nitrogen deficiency in *P. tricornutum* at various levels [13–17], no studies have been conducted on signal sensing and transduction or their regulatory pathways. In this study, we characterized the proteome, and metabolome of *P. tricornutum* under normal (NC), low nitrogen (LN) and high CO_2_ (HC) conditions. Western blotting was performed to assess lactylation in proteins. Lactylated proteomic analysis for NC was conducted, revealing the probable roles of lactylation in *P. tricornutum*. The results reveal that lactate might play an important role in C:N balance regulation and energy homeostasis maintaining in *P. tricornutum* through signal transduction as well as protein lactylation modification.

## Results

### Change in intracellular metabolite pools in response to HC and LN conditions

Table 1 shows the ratios of key cellular metabolites detected by NMR under different culture conditions (NC, LN and HC). In total, 41 metabolites, including alcohols (1), amines (2), amino acids and their derivatives (23), ammonium compounds (3), food and drug compounds (1), nucleic acid components (3), organic acids (7) and sugar (1), were identified. Most metabolites in LN and HC cultures showed similar regulation patterns, among which lactate was the most significantly upregulated metabolite, under both LN and HC conditions, compared to the NC condition, while 2-OG ranked second and third among significantly upregulated metabolites under LN and HC conditions, respectively. Of the 23 amino acids and their derivatives, the levels of two (Phe and Tyr) did not change; the levels of two (Gly and sarcosine) were increased, and the levels of the remaining 19 were decreased under LN and HC conditions, indicating N depletion. The levels of Gly and sarcosine were increased, indicating their possible importance in N redistribution. The levels of all the nucleic acid components detected were decreased, confirming N depletion. The levels of most organic acids were decreased, while increases in 2-OG and lactate reflected their important roles in C:N balance regulation. Glucose levels increased, likely because of increased carbohydrate catabolism. Some metabolites showed opposite expression patterns under HC and LN conditions. The levels of two amines (dimethylamine and methylamine) were increased under LN conditions and decreased under HC conditions, while the level of one ammonium compound (choline) decreased under LN conditions and increased under HC conditions.

**Table 1:** Ratios of abundances of key cellular metabolites under different culture conditions determined by NMR

Classification	Metabolites	LN/NC	P value	HC/NC	P value
Alcohols	myo-Inositol	1.160	0.077	2.464^**^	0.003
Amines	Dimethylamine	1.173^*^	0.032	0.384^**^	0.000
	Methylamine	1.790^**^	0.000	0.667^*^	0.013
Amino acid derivatives	Anserine	0.258^*^	0.014	0.260^**^	0.001
	Guanidoacetate	0.000^**^	0.003	0.000^**^	0.003
Amino acids	Aspartate	0.000^**^	0.000	0.000^**^	0.000
	Glutamine	0.000^**^	0.000	0.000^**^	0.000
	Methionine	0.000^**^	0.000	0.000^**^	0.000
	Serine	0.000^**^	0.000	0.000^**^	0.000
	Threonine	0.000^**^	0.000	0.000^**^	0.000
	Asparagine	0.000^**^	0.000	0.084^**^	0.000
	Arginine	0.831	0.265	0.089^**^	0.000
	Ornithine	0.011^**^	0.000	0.095^**^	0.000
	Proline	0.000^**^	0.000	0.171^**^	0.000
	Alanine	0.120^**^	0.000	0.286^**^	0.000
	Isoleucine	0.362^**^	0.000	0.348^**^	0.000
	Glutamate	0.124^**^	0.000	0.384^**^	0.000
	Leucine	0.393^**^	0.000	0.474^**^	0.000
	Valine	0.284^**^	0.000	0.491^**^	0.000
	Glycine	2.440^**^	0.000	4.487^**^	0.000
	Betaine	0.413^**^	0.000	0.672^**^	0.001
	Sarcosine	1.315^**^	0.000	1.288^**^	0.002
	Tyrosine	0.811^*^	0.015	0.785^**^	0.007
	Tryptophan	1.033	0.439	1.143	0.209
	Carnitine	0.536^*^	0.035	0.840	0.296
	Phenylalanine	1.250	0.174	1.004	0.490
Ammoniums compounds	Choline	0.124^*^	0.013	12.080^**^	0.000
	O-Phosphocholine	1.732^**^	0.005	1.417	0.205
	sn-Glycero-3-phosphocholine	1.183	0.230	0.863	0.384
Food and drug compounds	Trigonelline	0.806	0.104	1.063	0.346
Nucleic acid components	Inosine	0.000^**^	0.000	0.000^**^	0.000
	AMP	0.000^**^	0.000	0.245 ^**^	0.000
	NAD +	0.489^**^	0.002	0.664^**^	0.000
Organic acids	Propionate	0.088^**^	0.000	0.466^**^	0.000
	Lactate	40.517^**^	0.000	56.867^**^	0.000
	Acetate	0.136 ^**^	0.000	0.605^**^	0.002
	2-Oxoglutarate	14.112^**^	0.000	10.786^**^	0.002
	Ascorbate	0.409^**^	0.002	0.485^**^	0.004
	Succinate	0.601^**^	0.000	0.712^*^	0.023
	Fumarate	0.698	0.067	0.843	0.101
Sugars	Glucose	2.087^**^	0.000	3.903^**^	0.000

**Student t test P value < 0.01, *Student t test P value < 0.05, − Divide by zero

### Differential expression of proteins under HC and LN conditions

#### Overview of the proteomic data

Based on label-free proteome quantification technology, 5958 proteins were identified, among which 5325 proteins were quantified in *P. tricornutum* under NC, LN and HC conditions. Most peptides consisted of 7–20 amino acids (Additional file 6: Fig. S1a), which conforms to general rules based on enzymatic hydrolysis and mass spectrometry fragmentation. Most proteins corresponded to more than two peptides (Additional file 6: Fig. S1b). The coverage of most proteins was less than 30% (Additional file 6: Fig. S1c). The molecular weights of the identified proteins were evenly distributed (Additional file 6: Fig. S1d). A full range of functional annotations of the identified proteins was obtained on the basis of Gene Ontology (GO), protein domain, KEGG pathway, COG functional classification and subcellular structure positioning. The details of the annotations results are presented in Additional file 2: Table S1.

#### Identification and enrichment analysis of differentially expressed proteins (DEPs)

To understand the proteome responses to C:N ratio fluctuation in *P. tricornutum*, proteomic analysis based on label-free proteome quantification technology was performed to identify the DEPs in *P. tricornutum* under NC, LN and HC conditions. According to the criteria of a 1.5-fold increase (or 0.67-fold decrease) and a P value < 0.05 [24], a total of 1471 DEPs, including 694 differentially upregulated proteins (DUPs) and 777 differentially downregulated proteins (DDPs), were identified under LN conditions (Additional file 3: Table S2), and 327 DEPs, including 215 DUPs and 112 DDPs, were identified under HC conditions (Additional file 4: Table S3).

**Fig. 1:**
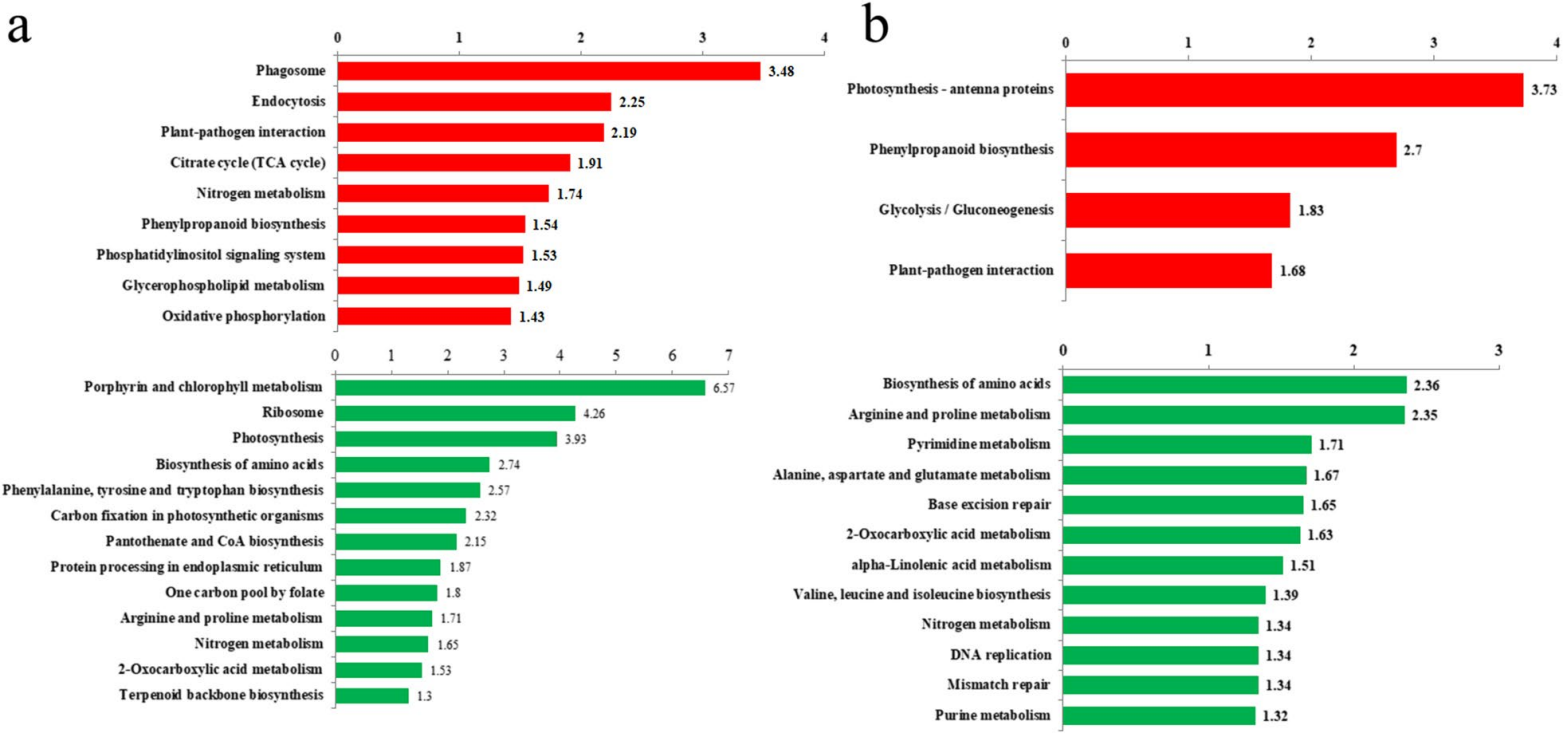
Enriched KEGG pathways analysis of differentially expressed proteins (all P < 0.05). **a** LN/NC. **b** HC/NC. Red, pathways terms of upregulated proteins. Green, pathways terms of downregulated proteins. X axis, − log10 (Fisher' exact test P value)

The KEGG pathway-based enrichment analysis revealed significant enrichment of DEPs in pathways involved in carbon metabolism, nitrogen metabolism, lipid metabolism, protein and amino acids metabolism, energy metabolism and signalling, including the enrichment of DUPs under LN conditions in TCA cycle, nitrogen metabolism, phenylpropanoid biosynthesis, phosphatidylinositol signalling system, glycerophospholipid metabolism and oxidative phosphorylation (Fig. 1a); the enrichment of DDPs under LN conditions in photosynthesis, biosynthesis of amino acids, carbon fixation in photosynthetic organisms and nitrogen metabolism (Fig. 1a), the enrichment of DUPs under HC conditions in photosynthesis—antenna proteins, phenylpropanoid biosynthesis and glycolysis / gluconeogenesis (Fig. 1b), and the enrichment of DDPs under HC conditions in biosynthesis of amino acids, pyrimidine metabolism and nitrogen metabolism (Fig. 1b).

#### Similar expression patterns of DEPs under LN and HC conditions indicated their functions in C:N balance regulation

As showed in Table 2, the third most upregulated DEP was predicted to be a transcription factor (UniProt B7G9R3), with fold changes of 44.9 and 17.1 under LN and HC conditions, respectively. The two most downregulated DEPs under LN conditions, UniProt B7G9B2 (0.0024-fold change) and UniProt B7G9B0 (0.0062-fold change), are two cell surface proteins and were found to be the second and fourth most downregulated proteins under HC conditions with a 0.0192-fold change and 0.0296-fold change, respectively. The third and fourth most downregulated DEPs under LN conditions, UniProt B7G9B1 (0.0115-fold change) and UniProt B7FYL2 (0.0145-fold change), are both activated upon iron starvation [7]. They were also downregulated under HC conditions, ranking first and seventh among DDPs with a 0.0114-fold change and 0.0859-fold change, respectively. Many DEPs (20 under LN and 2 under HC conditions) are members of the solute carrier family (Additional file 3: Table S2 and Additional file 4: Table S3), indicating a frequent exchange of metabolites. In addition, many DEPs are transcription factors or involved in signal transduction (Additional file 3: Table S2 and Additional file 4: Table S3), indicating that in *P. tricornutum,* the C:N balance might be regulated through signalling pathways. This similar expression pattern of DEPs under LN and HC conditions indicated they might play a role in C:N balance regulation.

**Table 2:** Different expressed proteins under LN/NC and HC/NC

Pathway	No.^*^	Protein accession	KEGG gene	HC/NC ratio	HC/NC P value	LN/NC ratio	LN/NC P value
Calvin cycle	36	A0T0E2	Ribulose-bisphosphate carboxylase small chain	0.69	0.21	0.47	0.03
	37	B5Y5F0	Phosphoribulokinase	0.97	0.72	0.47	0.00
	38	B7FRD1	Sedoheptulose-bisphosphatase	2.60	0.13	1.83	0.02
	39	B7FUH0	Alanine transaminase	0.63	0.10	0.54	0.04
	40	B7G9Y7	Aspartate aminotransferase	0.67	0.18	0.51	0.04
	50	B7G585	Pyruvate, orthophosphate dikinase	0.48	0.02	0.60	0.04
Calvin cycle, glycolysis	2	B7FSQ0	Triosephosphate isomerase	0.49	0.12	0.33	0.00
	3	B7FSI3	Glyceraldehyde 3-phosphate dehydrogenase	0.79	0.13	0.57	0.00
	5	B7G938	Phosphoglycerate kinase	0.79	0.11	0.69	0.00
	8	B7FSI4	Glyceraldehyde 3-phosphate dehydrogenase	0.72	0.16	0.50	0.01
	19	B7G5G4	Phosphoglycerate kinase	0.80	0.23	0.66	0.04
	21	B7G7C5	Glyceraldehyde 3-phosphate dehydrogenase	-	-	3.11	0.05
	23	B7GC94	Glyceraldehyde 3-phosphate dehydrogenase	1.56	0.04	1.50	0.05
Calvin cycle, glycolysis, OPPP	1	B7GE67	Fructose-bisphosphate aldolase	0.23	0.01	0.13	0.00
	6	B7G9G9	Fructose-bisphosphate aldolase	0.72	0.15	0.65	0.01
	12	B7G4R3	Fructose-bisphosphate aldolase	2.78	0.06	3.32	0.02
Calvin cycle, OPPP	29	B7FUU0	Transketolase	0.60	0.18	0.34	0.02
	31	B7FRG3	Ribulose-phosphate 3-epimerase	1.16	0.44	0.49	0.03
Calvin cycle, TCA cycle	30	B7GEG9	Malate dehydrogenase	1.05	0.82	1.50	0.01
Glycolysis	9	B7GD69	Probable phosphoglycerate mutase	1.85	0.07	2.51	0.01
	11	B7G9G7	Pyruvate kinase	0.64	0.15	0.41	0.02
	16	B7FNZ8	Alcohol dehydrogenase (NADP +)	0.94	0.69	1.67	0.03
	18	B7G8B7	Glucokinase	1.97	0.12	2.65	0.04
	20	B7FZG7	Pyruvate kinase	1.81	0.13	2.01	0.04
	22	B7FP91	S-(hydroxymethyl) glutathione dehydrogenase / alcohol dehydrogenase	0.74	0.11	1.44	0.05
	24	B7FRD3	Fructose-1,6-bisphosphatase I	0.71	0.06	0.75	0.07
	26	B7GA17	Glucose-6-phosphate 1-epimerase	1.97	0.02	1.21	0.16
	27	B7FT40	Alcohol dehydrogenase (NADP +)	0.62	0.05	0.76	0.18
Glycolysis, OPPP	4	B7GE27	6-Phosphofructokinase 1	0.50	0.09	0.28	0.00
	14	B7GCG9	6-phosphofructokinase 1	2.58	0.11	3.58	0.02
	15	B7GE28	6-Phosphofructokinase 1	1.37	0.44	1.99	0.03
Glycolysis, TCA cycle	7	B7S3L5	Pyruvate dehydrogenase E2 component (dihydrolipoamide acetyltransferase)	1.17	0.01	0.81	0.01
	10	B7GBE9	Dihydrolipoamide dehydrogenase	2.77	0.03	3.10	0.02
	13	B7G3I7	Pyruvate dehydrogenase E2 component (dihydrolipoamide acetyltransferase)	1.11	0.45	1.44	0.02
	17	B7FZN6	Pyruvate dehydrogenase E1 component beta subunit	1.34	0.14	1.58	0.03
	25	B7FZE1	Pyruvate dehydrogenase E1 component alpha subunit	4.16	0.00	1.72	0.08
	28	B7GDA9	Pyruvate dehydrogenase E2 component (dihydrolipoamide acetyltransferase)	1.44	0.05	1.16	0.22
Lactate metabolism	–	B7FZP8	D-2-hydroxyglutarate dehydrogenase	0.83	0.36	0.97	0.95
	–	B7G085	Lactoylglutathione lyase	0.97	0.98	0.73	0.27
	–	B7GDI1	Hydroxyacylglutathione hydrolase	1.28	0.19	1.31	0.10
	–	B7GDI2	Sulphur dioxygenase	1.51	0.21	1.51	0.11
Light-harvesting complex	62	B7G8E5	Light-harvesting complex I chlorophyll a/b binding protein 1	1.80	0.00	0.47	0.00
	63	B7G4U8	Light-harvesting complex I chlorophyll a/b binding protein 1	1.73	0.36	3.36	0.01
	64	B7GBK7	Light-harvesting complex I chlorophyll a/b binding protein 1	1.65	0.22	0.41	0.01
	65	B7FPL6	Light-harvesting complex I chlorophyll a/b binding protein 1	1.95	0.05	0.44	0.02
	66	B7GCV9	Light-harvesting complex I chlorophyll a/b binding protein 1	1.96	0.14	2.01	0.04
	67	B7FRW2	Light-harvesting complex I chlorophyll a/b binding protein 1	1.42	0.24	0.56	0.04
	68	B7G502	Light-harvesting complex I chlorophyll a/b binding protein 1	1.69	0.03	0.64	0.08
	69	B7GAS4	Light-harvesting complex I chlorophyll a/b binding protein 1	1.92	0.04	0.67	0.19
	70	B7G503	Light-harvesting complex I chlorophyll a/b binding protein 1	2.20	0.04	0.67	0.28
	71	B7FV42	Light-harvesting complex I chlorophyll a/b binding protein 1	2.38	0.04	1.02	0.75
Nitrogen metabolism	51	B7FNU0	Carbonic anhydrase	0.58	0.13	0.34	0.01
	52	B7FRE8	Cyanate lyase	0.99	0.91	0.53	0.05
	53	B7FXP8	Carbonic anhydrase	0.69	0.22	0.44	0.02
	54	B7FYS6	Formamidase	1.48	0.31	2.67	0.02
	55	B7FZB0	Glutamate synthase (NADH)	1.45	0.16	2.05	0.02
	56	B7G0L4	Ferredoxin-nitrite reductase	0.20	0.03	0.11	0.00
	57	B7G3X3	Glutamate dehydrogenase (NADP +)	1.29	0.54	1.76	0.03
	58	B7G5A1	Glutamine synthetase	2.56	0.01	3.14	0.00
	59	B7G997	Nitrate reductase (NAD(P)H)	0.09	0.00	0.19	0.02
	60	B7GA80	Carbonic anhydrase	0.38	0.14	0.33	0.02
	61	B7GAZ5	Glutamate synthase (NADH)	1.31	0.22	2.47	0.00
Oxidative phosphorylation	93	B7FVX2	NADH dehydrogenase (ubiquinone) 1 alpha subcomplex subunit 12	1.09	0.63	0.60	0.03
Oxidative phosphorylation	94	B7FRC2	NADH dehydrogenase (ubiquinone) 1 alpha/beta subcomplex 1, acyl-carrier protein	0.76	0.11	0.26	0.00
	95	B7GES5	NADH dehydrogenase (ubiquinone) Fe-S protein 7	1.14	0.47	1.79	0.02
	96	B7G964	NADH dehydrogenase (ubiquinone) flavoprotein 2	0.99	0.82	1.35	0.01
	98	B7G063	Ubiquinol-cytochrome c reductase subunit 7	0.99	0.99	0.56	0.01
	99	B7G3I2	V-type H + -transporting ATPase subunit a	1.18	0.32	2.68	0.00
	100	B7G162	V-type H^+^-transporting ATPase subunit A	1.04	0.80	1.58	0.04
	101	B7FQQ8	V-type H^+^-transporting ATPase subunit B	1.36	0.23	2.01	0.00
	102	B7FTS7	V-type H^+^-transporting ATPase subunit C	1.77	0.08	2.87	0.00
	103	B7G360	V-type H^+^-transporting ATPase subunit D	1.18	0.52	1.73	0.01
	104	B7G7X7	V-type H^+^-transporting ATPase subunit E	1.47	0.14	2.02	0.00
	105	B7G9S7	V-type H^+^-transporting ATPase subunit H	1.42	0.27	2.86	0.00
	106	B7GE53	H^+^-transporting ATPase	1.04	0.90	1.97	0.02
	107	B7FT09	inorganic pyrophosphatase	0.92	0.36	0.58	0.00
	97	B7GDY0	V-type H^+^-transporting ATPase subunit F	1.48	0.04	0.93	0.17
Photosynthesis	72	A0T0A3	Photosystem II cytochrome b559 subunit alpha	1.16	0.70	0.30	0.00
	73	A0T0A9	Photosystem II PsbH protein	1.17	0.68	0.27	0.00
	74	A0T0B2	Photosystem II CP47 chlorophyll apoprotein	0.91	0.58	0.69	0.01
	75	A0T0C6	Photosystem II cytochrome c550	0.89	0.60	0.27	0.00
	76	B7FUR5	Photosystem II PsbU protein	0.71	0.35	0.16	0.00
	77	B7FZ96	Photosystem II oxygen-evolving enhancer protein 1	0.77	0.30	0.27	0.00
	78	B7G9T8	Ferredoxin–NADP^+^ reductase	1.17	0.48	1.60	0.03
	79	B7GCT8	Ferredoxin-NADP^+^ reductase	0.63	0.15	0.40	0.01
	80	A0T0B8	Cytochrome b6	1.20	0.15	1.59	0.01
	81	B5Y578	Cytochrome c6	0.80	0.47	0.28	0.00
	82	B5Y3C9	Cytochrome b6-f complex iron-sulphur subunit	1.68	0.00	0.67	0.02
	83	A0T0M6	Photosystem I subunit XI	2.11	0.02	0.88	0.66
	84	A0T0B9	Photosystem I subunit II	1.01	0.96	0.48	0.01
	85	A0T0F3	Photosystem I subunit IV	0.91	0.60	0.42	0.00
	86	A0T0L2	Photosystem I subunit VII	1.24	0.19	0.35	0.00
Photosynthesis, Oxidative phosphorylation	87	A0T0D1	F-type H^+^/Na^+^-transporting ATPase subunit beta	0.86	0.54	0.39	0.01
	88	A0T0D2	F-type H^+^/Na^+^-transporting ATPase subunit beta	0.85	0.44	0.50	0.01
	89	A0T0F0	F-type H^+^-transporting ATPase subunit delta	1.44	0.10	0.66	0.01
	90	A0T0F1	F-type H^+^/Na^+^-transporting ATPase subunit alpha	0.82	0.38	0.54	0.02
	91	B7G0M9	F-type H^+^-transporting ATPase subunit gamma	0.74	0.23	0.44	0.01
	92	B7FRE6	F-type H^+^/Na^+^-transporting ATPase subunit alpha	0.77	0.01	0.77	0.01
OPPP	32	B5Y3S6	transaldolase	1.44	0.15	1.51	0.00
	33	B7FPL4	6-Phosphogluconolactonase	1.26	0.50	1.53	0.05
	34	B7FQV1	rRbose-phosphate pyrophosphokinase	0.97	0.72	0.67	0.01
	35	B7GDN8	Transaldolase	0.97	0.75	0.37	0.00
TCA cycle	42	B7FUR4	Aconitate hydratase 2 / 2-methylisocitrate dehydratase	1.81	0.06	2.54	0.00
	43	B7FWX5	Fumarate hydratase, class I	1.55	0.10	2.11	0.00
	44	B7FXA2	Succinyl-CoA synthetase beta subunit	1.18	0.16	1.47	0.00
	45	B7G4T8	2-Oxoglutarate dehydrogenase E1 component	1.41	0.25	2.03	0.05
	46	B7G620	Isocitrate dehydrogenase	1.21	0.54	1.86	0.03
	47	B7G9P5	Citrate synthase	1.60	0.10	1.81	0.00
	48	B7GA05	Phosphoenolpyruvate carboxykinase	0.72	0.01	0.51	0.00
	49	B7GA98	Pyruvate carboxylase	0.67	0.01	1.04	0.68
TCA cycle, Oxidative phosphorylation	41	B5Y4R0	Succinate dehydrogenase (ubiquinone) cytochrome b560 subunit	1.86	0.11	2.95	0.01
Chrysolaminarin metabolism	–	B7FWJ8	Beta-glucosidase	1.91	0.01	1.64	0.01
	–	B7GB76		1.69	0.09	3.26	0.01
	–	B7GBX3	Xylan 1,4-beta-xylosidase	1.31	0.54	2.14	0.03
Phosphatidylinositol signaling system	–	B7FSY9	Inositol polyphosphate 5-phosphatase INPP5B/F	1.03	0.79	1.90	0.04
	–	B7G377	1-Phosphatidylinositol-4-phosphate 5-kinase	1.17	0.32	1.81	0.00
	–	B7G4M3	Diacylglycerol kinase (ATP)]	3.46	0.11	5.70	0.00
	–	B7GBU7	Phosphatidylinositol phospholipase C, delta	1.40	0.06	2.53	0.00
	–	B7GCH9	Phosphatidylinositol 4-kinase B	1.24	0.47	2.29	0.02
	–	B7GD06	Calmodulin	1.73	0.00	1.61	0.00
Other proteins showing similar expression patterns under LN and HC	–	B7G9R3	–	17.07	0.01	44.89	0.00
	–	B7G9B2	–	0.02	0.03	0.00	0.00
	–	B7G9B0	–	0.03	0.00	0.01	0.00
	–	B7G9B1	–	0.01	0.01	0.01	0.01

^*^Protein No. in Fig. 3

#### DEPs involved in phenylpropanoid biosynthesis and nitrogen metabolism

**Fig. 2:**
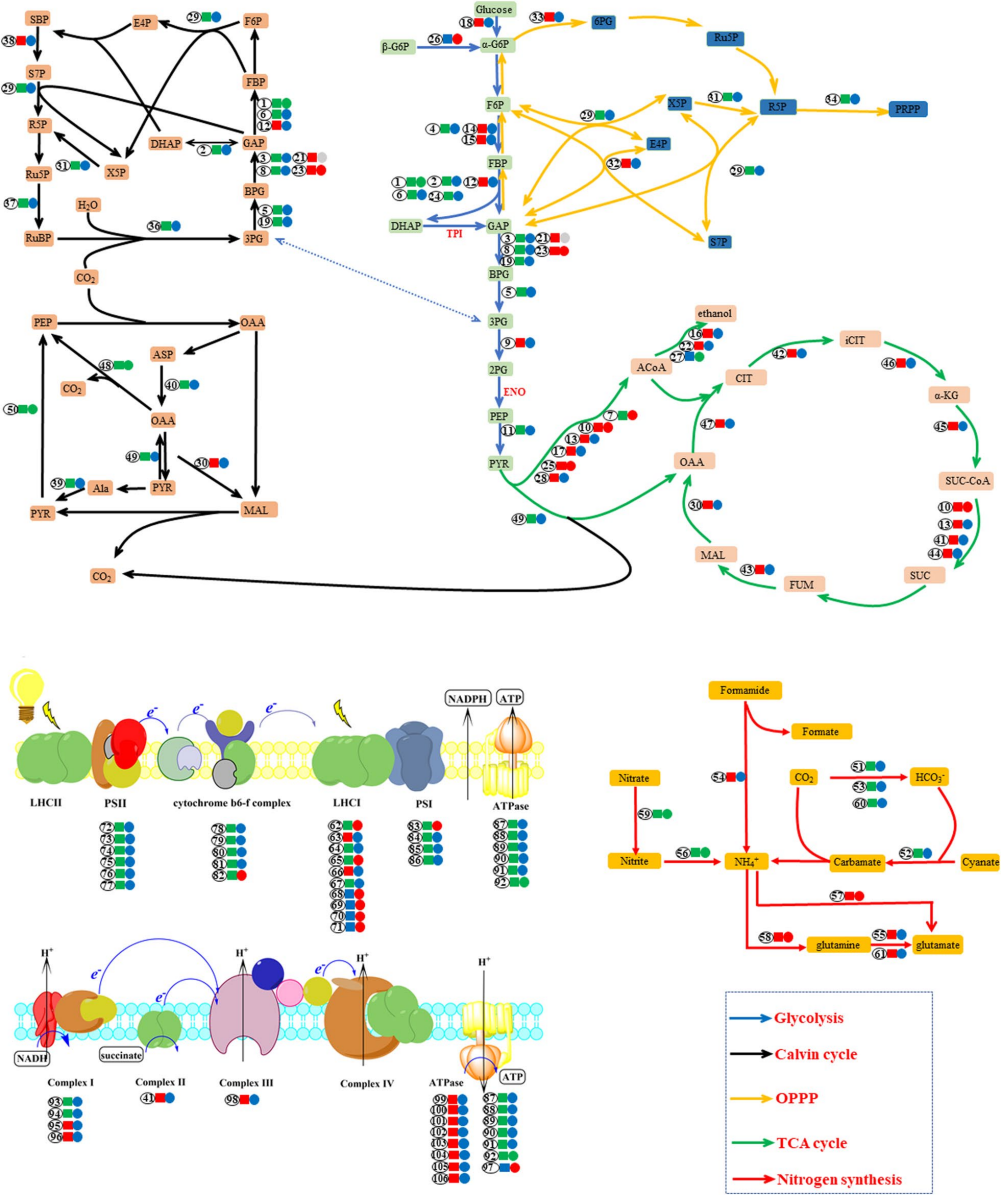
Different expressed proteins involved in photosynthesis, carbon concentration mechanism (CCM), glycolysis, Calvin cycle, TCA cycle, pentose phosphate pathway, and oxidation phosphorylation. The number in the circle indicates the no. of proteins listed in Table 2 where the full information for the proteins can be obtained. Square, LN/NC; circle, HC/NC. Red, upregulation of protein content. Green, downregulation of protein contents. Blue, the regulation mood of protein content. Grey, proteins do not detect in the experiment group (LN or HC)

The most upregulated DEP under both LN (790.8-fold change) and HC (287.8-fold change) was predicted to be peroxiredoxin (Tables S2 & S3), which is involved in phenylpropanoid biosynthesis. The second and fifth (LN) or fourth (HC) most upregulated DEPs were predicted to be transcription factors (UniProt B7GDM6 and UniProt B7FUS4) involved in the regulation of phenylpropanoid biosynthesis, with fold changes of 167.6 and 26.4 under LN conditions and 50.0 and 14.4 under HC conditions (Tables S2 & S3), respectively. A total of 11 DEPs were involved in nitrogen metabolism under LN, while those of HC were three (Table 2, Fig. 2), indicating that though *P. tricornutum* experienced nitrogen deficiency stress under both LN and HC conditions, it is more severe under LN.

#### DEPs involved in photosynthesis, carbon concentration mechanism (CCM), central carbon metabolism and chrysolaminarin degradation

Two DUPs and four DDPs in LN were light-harvesting complex-related proteins. A total of 18 DDPs and two DUPs under LN were involved in photosynthesis, indicating a severe downregulated of photosynthesis under LN (Table 2, Fig. 2). Five light-harvesting complex-related proteins were upregulated under HC condition, indicating enhance of photosynthesis under HC (Table 2, Fig. 2). In addition, proteins involved in the CCM were significantly downregulated under LN conditions, including two Na^+^-independent C_l_/HCO_3_^−^ exchangers (UniProt B7S435 and B7S437); four carbonic anhydrases (CA, UniProt B7FNU0, B7GA80, B7FXP8, and B5Y401); pyruvate, orthophosphate dikinase (PPDK, UniProt B7G585); and phosphoenolpyruvate carboxykinase (PEPCK, UniProt B7GA05) (Table 2, Fig. 2). A CA (B7FNT2) and a PPDK (UniProt B7G585) were significantly downregulated under HC condition (Table 2, Fig. 2). Under LN, proteins involved in Calvin cycle, glycolysis, and pentose phosphate pathway were mainly downregulated, while that of TCA cycle increased (Table 2, Fig. 2). Under HC there were not significantly change in most proteins involved in the central carbon metabolism, except that some proteins involved in glycolysis increased. Diatoms store carbon in the form of 1,3-β-d-glucan (chrysolaminarin) or lipids. We observed an increase in β-glucosidase under both LN (UniProt B7GBX3, UniProt B7FWJ8 and UniProt B7GB76) and HC (UniProt B7FWJ8) conditions (Table 2), indicating the degradation of chrysolaminarin.

#### DEPs involved in phosphatidylinositol signalling system

Five proteins involved in phosphatidylinositol signalling system, including diacylglycerol kinase (DGK), 1-phosphatidylinositol-4-phosphate 5-kinase (PIP5K), phosphatidylinositol phospholipase C (PLCD), calmodulin, phosphatidylinositol 4-kinase B (PI4KB) and inositol polyphosphate 5-phosphatase (INPP5B/F) were all significantly up-regulated under LN condition (Table 2), indicating phosphatidylinositol signalling system might play an important role in nitrogen-limited response.

#### DEPs involved in lactate metabolism and lysine lactylation

D-lactate dehydrogenase (*ldhA*), which catalyses the dehydration of pyruvate to generate D-lactate, was upregulated under both LN (5.6-fold change) and HC (4.5-fold change) conditions, a finding consistent with lactate accumulation under both LN and HC conditions. Besides, a l-lactate permease (LCTP), which was reported to expressed under nitrogen-limited conditions [7], and enzymes related to D-lactylation, namely, lactolglutathione lyase (GLXI) and glyoxalase (GLO1 and GLO2) [25], were also detected in the *P. tricornutum* proteome analysis, although their levels did not vary significantly upon different treatments (Table 2).

### Protein validation by PRM and biochemical analysis

**Fig. 3:**
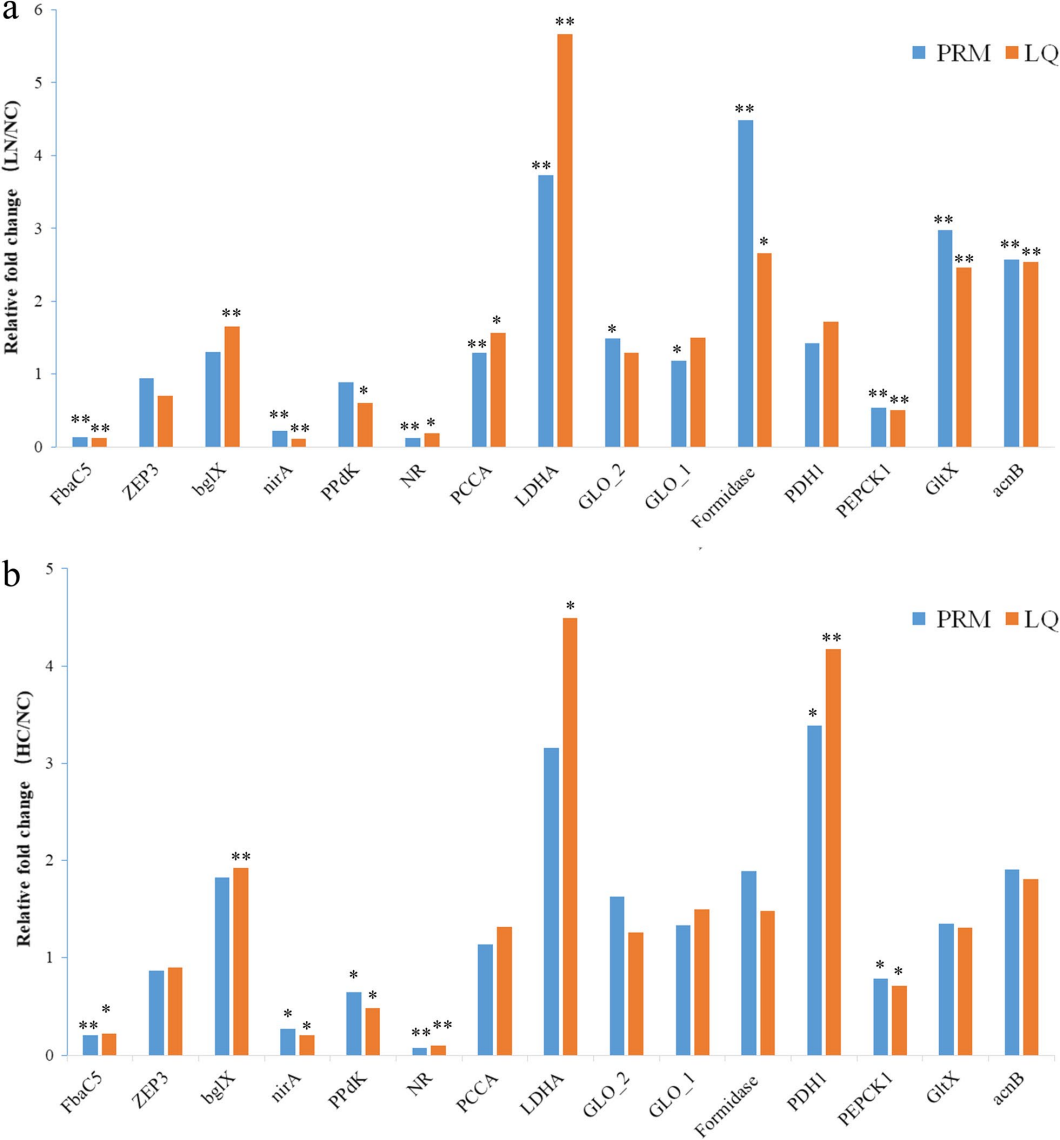
The comparison of protein expression by label free and PRM. **a** LN/NC. **b** HC/NC. *FbaC5* fructose-bisphosphate aldolase, *ZEP3* zeaxanthin epoxidase, *bglX* beta-glucosidase, *nirA* ferredoxin-nitrite reductase, *PPdK* pyruvate, orthophosphate dikinase, *NR* nitrate reductase, *PCCA* carboxylase propionyl-CoA carboxylase, *LDHA* D-lactate dehydrogenase, *GLO_2* glyoxalase OS, *GLO_1* glyoxalase, *PDH1* precursor of dehydrogenase pyruvate dehydrogenase E1, *PEPCK1* phosphoenolpyruvate carboxykinase, *GltX* ferredoxin-dependent glutamate synthase, *acnB* aconitate hydratase 2 / 2-methylisocitrate dehydratase

We performed PRM to eliminate most of the possible errors though the label-free data in this report had undergone rigorous statistical and bioinformatics analysis. Analysis on FbaC5, ZEP3, bglX, nirA, PPdK, NR, PCCA, LDHA, GLO_2, GLO_1, Formidase, PDH1, PEPCK1, GltX and acnBto was performed to validate the label-free results. The results of target proteins detected by PRM were consistent with the label-free analysis (Fig. 3), which indicated that our proteomics results based on label free were highly reliable and reproducible.

### Pan-acetylation and pan-lactyllysine Western blotting analyses

**Fig. 4:**
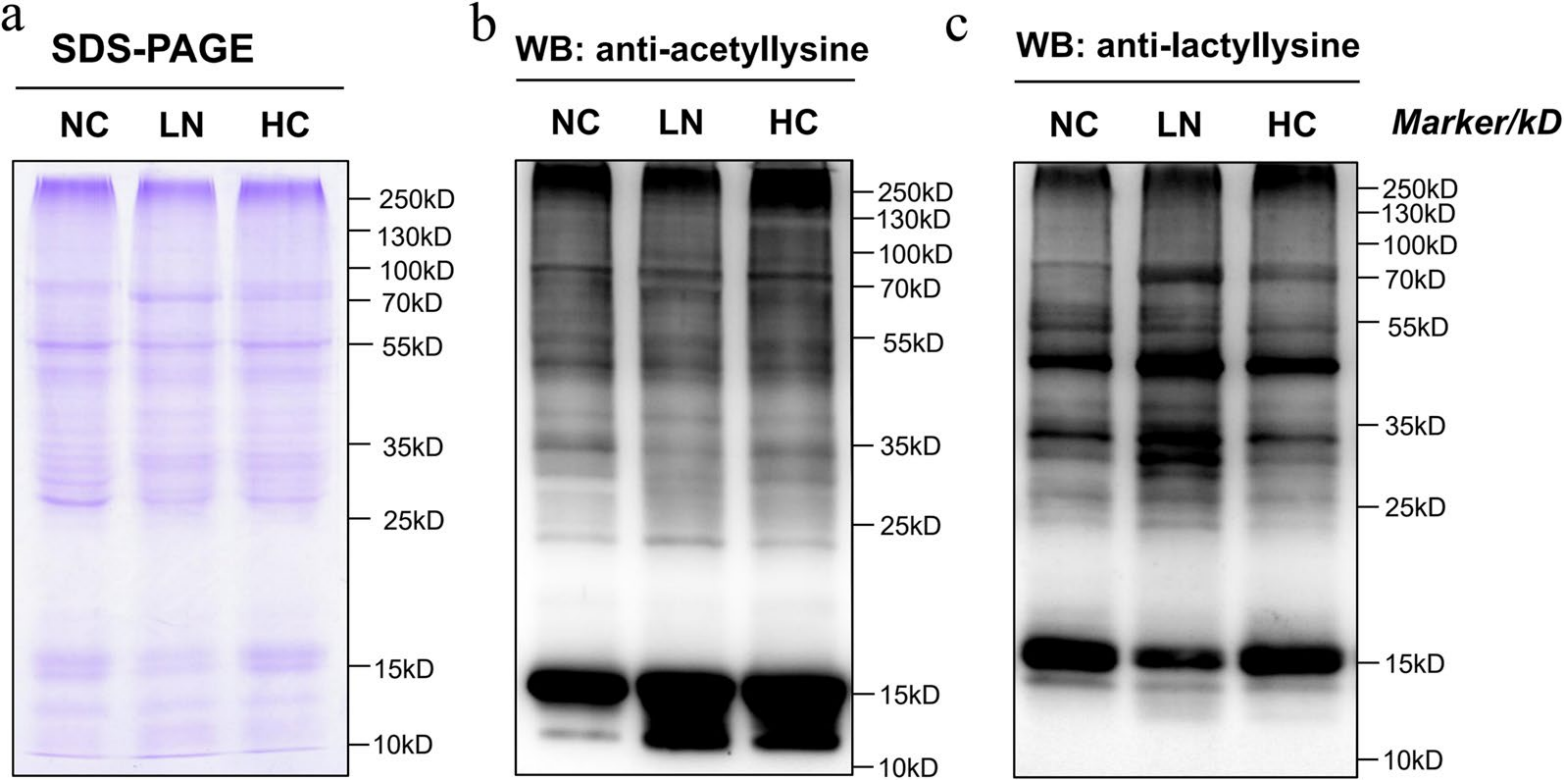
Pan-acetylation and pan-lactyllysine Western blotting analyses. **a** Coomassie brilliant blue staining. **b** Western blotting with pan anti-acetyllysine antibody. **c** Western blotting with pan anti-lactyllysine antibody

The overall regulation of acetylation and lactylation patterns of all proteins was determined by Western blotting using a pan anti-acetyllysine antibody and a pan-lactyllysine antibody, respectively. As shown in Fig. 4, many proteins were either acetylated or lactylated under NC, LN and HC conditions, but the overall acetylation and lactylation levels differed. Increased lactylation was observed on larger proteins under LN and HC conditions, consistent with the increase in lactate content implied by the metabolomic analysis results. Under LN and HC conditions, increased acetylation was observed on proteins with a molecular weight of 12–15 kDa, which were most likely members of the histone family. These findings confirmed that protein lactylation proceeds through temporal dynamics that differ from those of protein acetylation.

### Lactylated proteomic analysis under NC in *P. tricornutum*

#### Global profiling of lactylated proteome

**Fig. 5:**
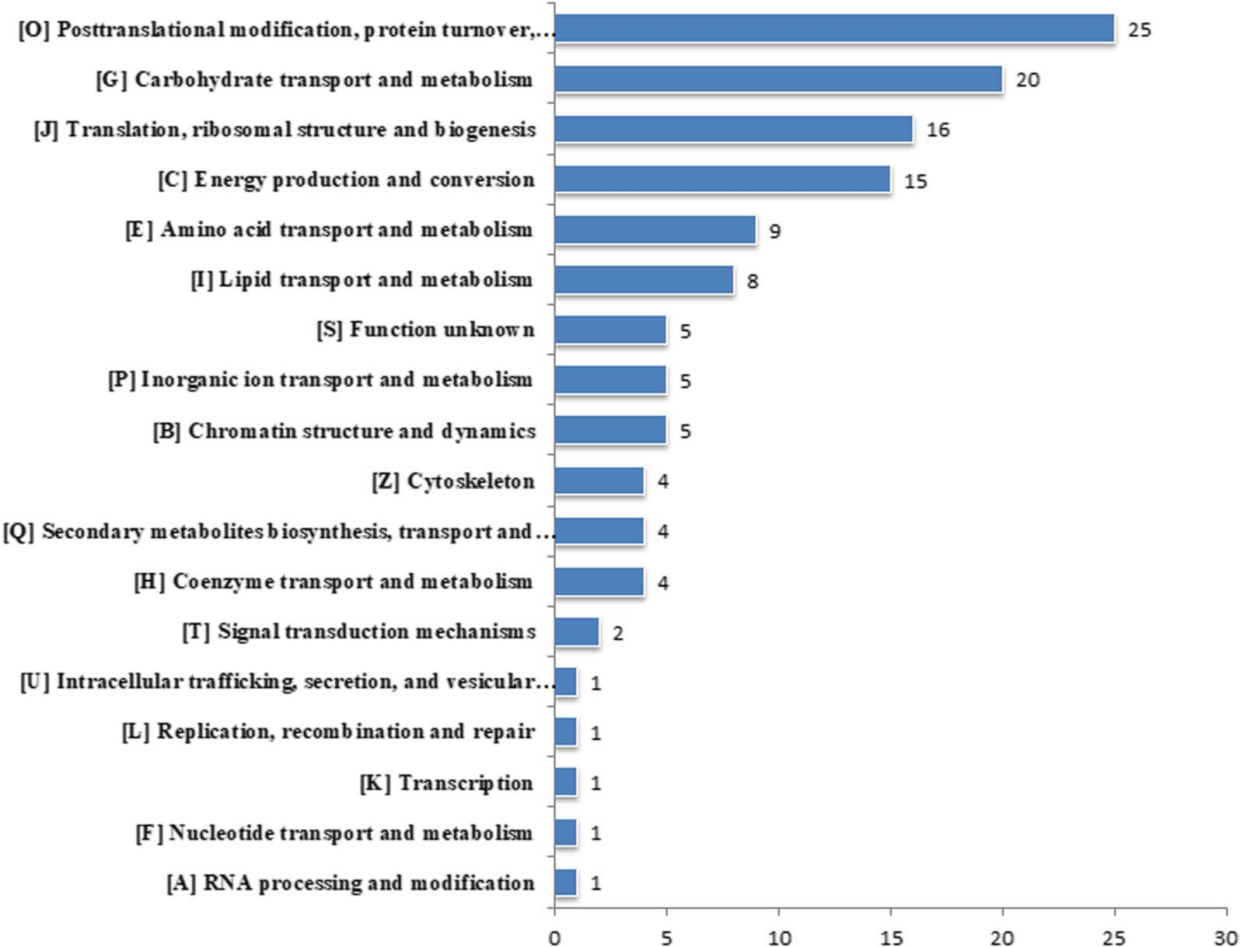
Clusters of Orthologous Groups (COG) annotation for lactylated proteins

To study the probable function of lysine lactylation in *P. tricornutum*, we performed global profiling of the lysine lactylome for NC. A total of 463 lactyllysine sites across 212 proteins were identified (Additional file 5: Table S4). To further understand the biological regulations and functions of lactylated proteins in C/N balance in *P. tricornutum*, we carried out Clusters of Orthologous Groups (COG) annotation and KEGG pathway enrichment analysis. The results of COG functional annotation analysis showed that various lactylated proteins were engaged with crucial biological processes, such as posttranslational modification, protein turnover and chaperones; carbohydrate transport and metabolism; translation, ribosomal structure and biogenesis; energy production and conversion; amino acid transport and metabolism; and lipid transport and metabolism (Fig. 5).

#### Lactylated proteins are enriched on photosynthesis, central carbon metabolism and fatty acid biosynthesis pathways

**Fig. 6:**
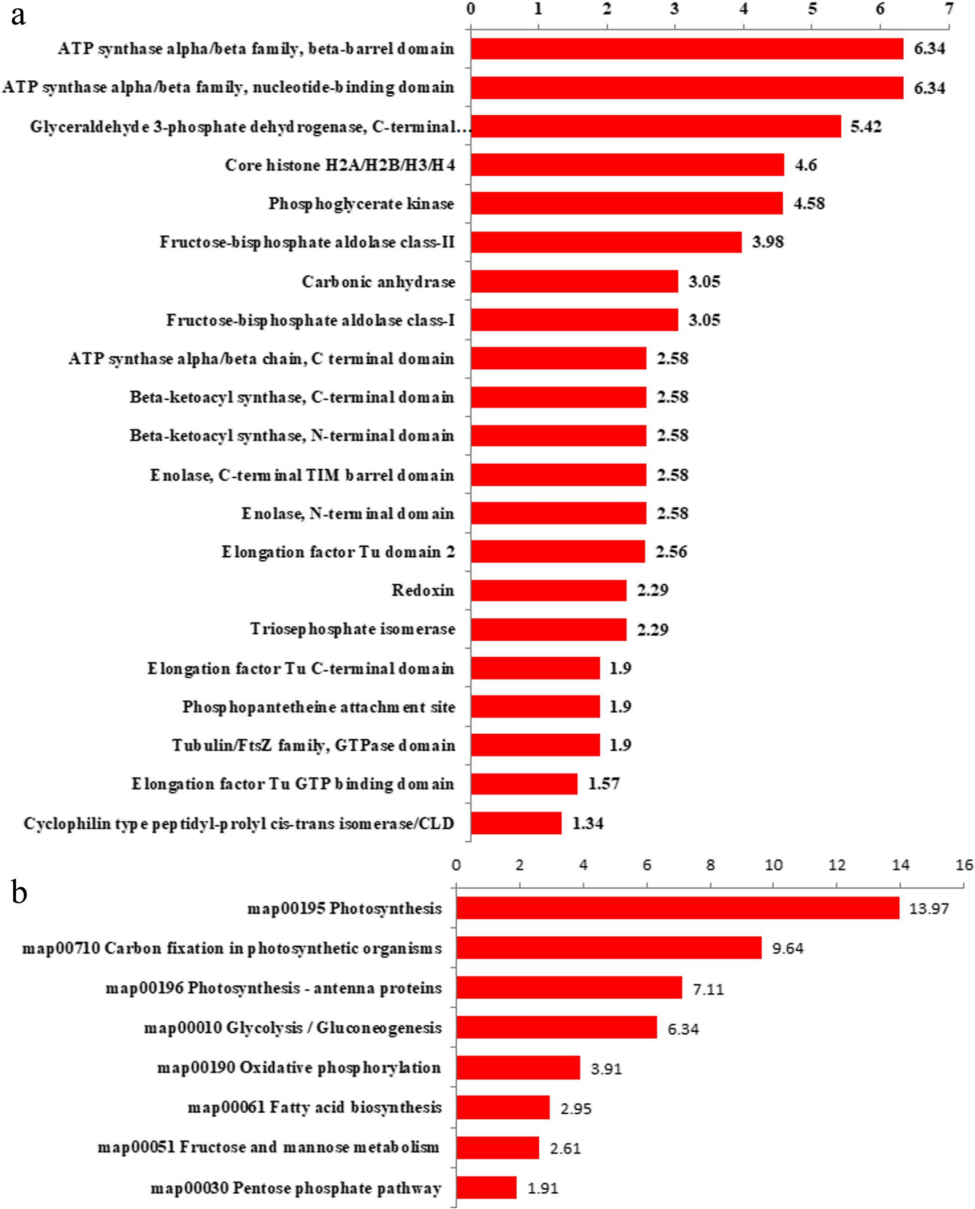
Protein domain enrichment analysis (**a**) and KEGG pathway enrichment analysis (**b**) for lactylated proteins

Protein domain analysis revealed that lactylated proteins were concentrated in ATP synthase, GADPH, core histone H2A/H2B/H3/H4, phosphoglycerate kinase, fructose-bisphosphate aldolase, carbonic anhydrase, enolase and so on (Fig. 6a). KEGG pathway enrichment analysis illustrated that lactylated proteins were concentrated in photosynthesis, carbon fixation in photosynthetic organisms, photosynthesis-antenna proteins, glycolysis/gluconeogenesis, oxidative phosphorylation, fatty acid biosynthesis, fructose and mannose metabolism and pentose phosphate pathway (Fig. 6b), indicating that lactylation played an important role in energy balance and carbon metabolism.

**Fig. 7:**
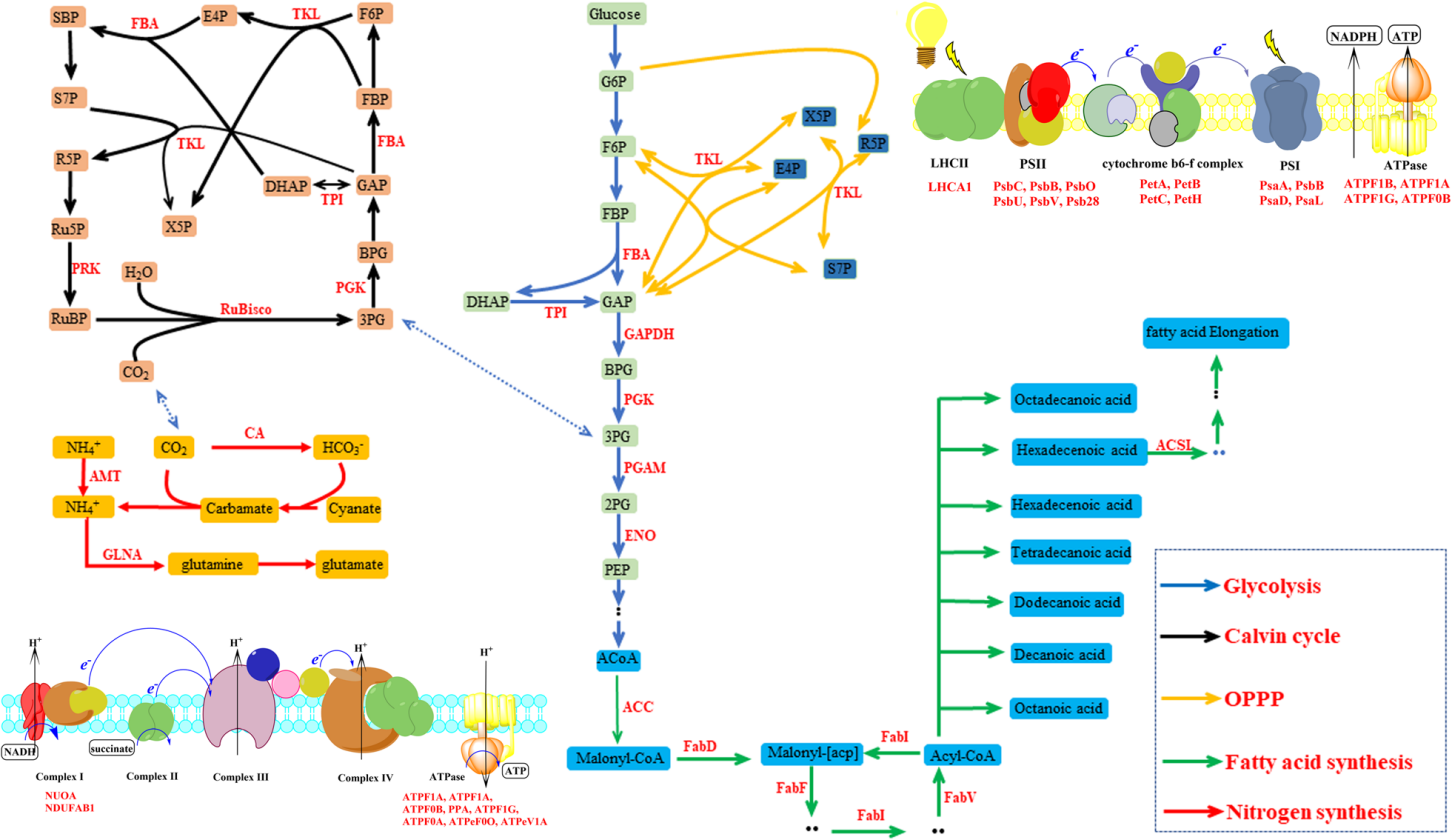
Lactylated proteins involved in photosynthesis, glycolysis, Calvin cycle, TCA cycle, pentose phosphate pathway, fatty acid synthesis and oxidation phosphorylation. *FBA* fructose-bisphosphate aldolase, *TPI* triosephosphate isomerase, *GAPDH* glyceraldehyde 3-phosphate dehydrogenase (phosphorylating), *GAP* d-glyceraldehyde 3-phosphate, *PGK* phosphoglycerate kinase, *BPG* 3-phospho-d-glyceroyl phosphate, *3PG* 3-phospho-d-glycerate, *2PG* phospho-d-glycerate, *PGAM* 2,3-bisphosphoglycerate-dependent phosphoglycerate mutase, *ENO* enolase, *X5P* d-xylulose 5-phosphate, *TKL* transketolase, *E4P* d-erythrose 4-phosphate, *S7P* sedoheptulose 7-phosphate, *SBP* sedoheptulose 1,7-bisphosphate, *PRK* phosphoribulokinase, *MCOA* malonyl-CoA, *ACACA* acetyl-CoA carboxylase / biotin carboxylase 1, *Malonyl-[acp]* malonyl-[acyl-carrier protein], *FabD* [acyl-carrier-protein] S-malonyltransferase, *ACSL* long-chain acyl-CoA synthetase, *FabF* 3-oxoacyl-[acyl-carrier-protein] synthase II, *FabI* enoyl-[acyl-carrier protein] reductase I, *CA* carbonic anhydrase, *AMT* ammonium transporter, *LHCA* light-harvesting complex I chlorophyll a/b binding protein 1, *ATPF1A* F-type H^+^/Na^+^-transporting ATPase subunit alpha, *ATPF1B* F-type H^+^/Na^+^-transporting ATPase subunit beta, *ATPF1G* F-type H^+^-transporting ATPase subunit gamma, *ATPF0B* F-type H^+^-transporting ATPase subunit b, *NUOA* NADH-quinone oxidoreductase subunit A, *NDUFAB1* NADH dehydrogenase (ubiquinone) 1 alpha/beta subcomplex 1, acyl-carrier protein, *ATPF0A* F-type H^+^-transporting ATPase subunit a, *PPA* inorganic pyrophosphatase, *ATPeF0O* F-type H + -transporting ATPase subunit O, *ATPeV1A* V-type H^+^-transporting ATPase subunit A

Lactylated proteins involved in glycolysis/gluconeogenesis, Calvin cycle, pentose phosphate pathway and fatty acid synthesis are shown in Fig. 7. Besides, nitrogen metabolism-related proteins, including ammonium transporter, glutamine synthetase and carbonic anhydrase, were also detected (Fig. 7), indicating that lactylation also played an important role in nitrogen metabolism. Simultaneously, components of oxidative phosphorylation and photosynthetic phosphorylation were also detected, indicating that lactylation participates in energy metabolism.

Interestingly, lactylation mainly occurred in proteins that located in cytoplasm and chloroplast, but rarely in proteins located in mitochondria, implying that protein lactylation modifications are affected by cell compartmentalization. It is probable that there is lactate permease on chloroplast membrane, but not on mitochondria membrane.

### Influence of lactate on growth and total lipid content of *P. tricornutum*

**Fig. 8:**
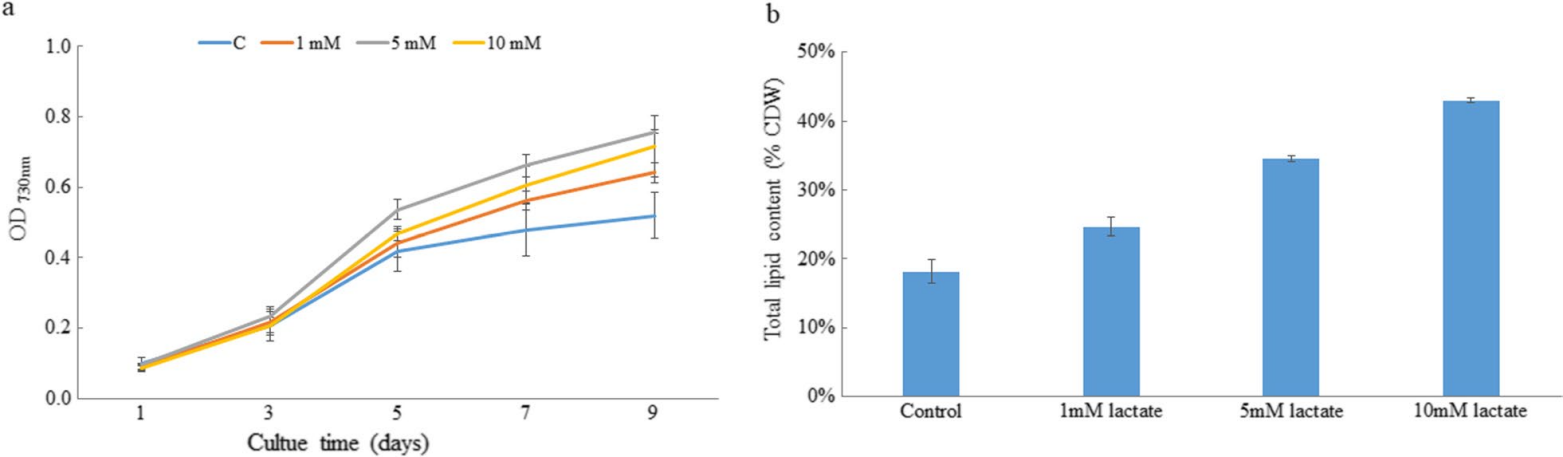
Influence of lactate on growth (**a**) and total lipid content (**b**) of *P. tricornutum*

As shown in Fig. 8, lactate at 1 mM, 5 mM and 10 mM could increase (P < 0.05) growth of *P. tricornutum*, with 5 mM being the most significant. Total lipid content was determined to verify if lactate influenced total lipid content. The results showed that lactate can significantly (P < 0.05) promote the accumulation of lipids in *P. tricornutum*, and the total lipid content increases with the increase of lactate concentration, indicating that lactate might play an important role in lipid metabolism in *P. tricornutum*. Yet as lactate can also function as a carbon source, further studies, such as ^13^C labelling, are needed to verify the function of lactate in *P. tricornutum*.

## Discussion

### 2-OG might regulate *P. tricornutum* C:N balance by suppressing CCM

In recent decades, the response of *P. tricornutum* to nitrogen deficiency has received considerable attention. Research mainly concerns the physiological response, gene expression and metabolic pathway shift or lipid accumulation process [12–14, 26]. However, the mechanisms behind *P. tricornutum* responses, e.g. sensing nitrogen deficiency and transmitting relevant signals to induce the response mechanism, have not been elucidated. To some extent, nitrogen deficiency (LN) and a high CO_2_ concentration (HC) indicate a high C:N ratio [23, 27], which might trigger the activation of a series of signalling pathways. For example, 2-OG indicates a high C:N ratio and plays an important role in the regulation of carbon and nitrogen metabolism in cyanobacteria [21]. By depressing the expression of genes involved in CCM, 2-OG represses carbon fixation and thus maintains the intracellular C:N balance. In this study, we observed 14-fold and 12-fold upregulation of 2-OG expression under LN and HC conditions (Table 1). In addition, proteins involved in the CCM were significantly downregulated under LN conditions (Table 2). The accumulation of 2-OG and the downregulation of CCM-related genes under both LN and HC conditions indicated that 2-OG might play an important role in sensing and transducing high C:N ratio signals and act as a ligand that interacts with carbon fixation regulators. This supposition is consistent with the idea that carbon metabolism induced in diatoms in response to nitrogen starvation bears closer resemblance to the response of cyanobacteria than that of higher plants or green algae [6].

### Probable role of lactate in *P. tricornutum* C:N balance regulation

In addition to an increase in 2-OG, we also observed a dramatic increase in lactate content under both LN and HC conditions. Lactate is used to be considered as a metabolism waste by-product of glycolysis. Currently, lactate is considered a crucial regulatory nexus for energy metabolism [28], a signalling molecule participating in a series of metabolic processes [29, 30] and a substrate for posttranslational modifications (PTMs) through the ‘LactoylLys’ modification of proteins [25, 31]. The increase in lactate under both LN and HC conditions indicated its likely roles in the following processes:

1. Regulation of energy balance. In organisms, the main source of ATP is oxidative phosphorylation, in which electrons in NADH (produced by cell metabolism) are delivered to oxygen via the respiratory chain with the concomitant production of ATP and NAD^+^. Under hypoxic conditions, oxidative phosphorylation is repressed, NADH accumulates and NAD^+^ regeneration is blocked. To maintain the energy supply under hypoxic conditions, plants switch from respiration to fermentative metabolism by activating lactate dehydrogenase, which catalyses the reduction of pyruvate to generate lactate, accompanied by the consumption of NADH. Thus, lactate dehydrogenase can regulate cellular energy balance. In this study, we observed a significant decrease in AMP under both LN and HC conditions (Table 1), indicating a high ATP:AMP ratio; e.g., cells are in high energy mode. This outcome might be a result of a decrease in the NADH consumption process, i.e. nitrogen reduction. We observed a decrease expression in nitrate reductase (UniProt B7G997) and ferredoxin-nitrite reductase (UnitProt B7G0L4) under both LN and HC conditions (Table 2, Fig. 2). These results might indicate decreased NADH consumption. Furthermore, processes in which NADH is produced, i.e., the TCA cycle under LN conditions and pyruvate dehydrogenase and photosynthesis under HC conditions, were accelerated. This acceleration might disrupt the energy balance and activate lactate dehydrogenase, resulting in the accumulation of lactate. In this study, we observed a 5.57-fold and 4.46-fold increase in lactate dehydrogenase (ldhA, B7S4E4) under LN and HC conditions (Table2), indicating the upregulation of lactate reductant from pyruvate. Moreover, the shuttling of L-lactate between different organs and cells creates a major circulatory mechanism of carbohydrate sources and NADH/NAD^+^ [32]. In this study, we also detected L-lactate permease (LCTP, Gene ID: 7,195,398) under LN condition (Table 2 and Additional file 2: Table S1), suggesting the important role of lactate in the C:N balance and energy balance regulation.
2. Regulation of lipid metabolism. In contrast to cyanobacteria, *P. tricornutum* is an oleaginous microalga that accumulates lipids under nitrogen-limited conditions. In this process, *P. tricornutum* remodels intracellular components and redistributes metabolites [14]. Therefore, its C:N balance regulation may not be limited to the assimilation process of carbon and nitrogen and may also be related to the degradation and redirection of intracellular catabolic products. Lactate itself can act as a signal molecule in the cAMP pathway and regulate a series of cellular processes. In mammalian adipose tissue, for example, lactate can combine with its sensor, an orphan G protein-coupled receptor, activating it and suppressing lipolysis in fat cells [30]. Despite its categorization as a photosynthetic organism, *P. tricornutum* has an unusual evolutionary history, with secondary endosymbiotic origin and mosaic genome that contains ‘animal-like’, ‘plant-like’ and ‘bacteria-like’ genes [33–37]. Some of its genes are more similar to those of animals than to their photosynthetic counterparts [38]. Recently, it was reported that in *P. tricornutum*, phosphatidylinositol 3-kinase (PI3K), which is involved in lipid accumulation, is more closely associated with mammalian homologues than those in higher plants [39]. The accumulation of both lactate and lipids in *P. tricornutum* under LN and HC conditions indicated that lactate might also suppress lipolysis in *P. tricornutum,* as it does in mammalian cells, and thus facilitate lipid accumulation in *P. tricornutum* under LN and HC conditions.
3. ‘LactoylLys’ modification on proteins (lactylation). Researchers recently found that lactate can regulate protein functions through posttranslational modifications (PTMs) by conjugating to protein Lys residues and generating a ‘LactoylLys’ modification on proteins, revealing non-metabolic functions of lactate [31]. Protein lactylation is a new topic currently studied mainly in mammals, as protein lactylation plays an important role in diseases, such as neoplasia, sepsis and autoimmune diseases. Studies related to protein lactylation in algae have not been reported. Protein lactylation is positively correlated with intracellular lactate concentration [25]. In this study, lactate was the most significantly upregulated metabolite under both LN and HC conditions. The WB results (Fig. 4) indicated proteins in *P. tricornutum* had undergone lactylation, which was particularly evident under LN and HC conditions. Lactylated proteomics under NC condition revealed that lactylated proteins were significantly involved in photosynthesis, oxidative phosphorylation, central carbon metabolism including Calvin cycle, glycolysis and pentose phosphate pathway, fatty acid synthesis and nitrogen metabolism, confirming protein lactylation in *P. tricornutum* played an important role in C:N balance regulation, energy homeostasis maintenance and fatty acid synthesis.

### Probable role of the IP3K signalling pathway under LN conditions

In this study, we found that genes involved in the phosphatidylinositol signalling system were upregulated at the protein level (Table 2) under LN conditions. It has been reported that in *P. tricornutum,* the expression of genes involved in MAPK signalling was upregulated, while that in lipid metabolism was decreased under treatment with a phosphatidylinositol 3-kinase inhibitor, demonstrating a role for phosphatidylinositol 3-kinase in the allocation of carbon, including as a factor of lipid reduction in *P. tricornutum *[39]. In a study of the green alga *Chlamydomonas reinhardtii*, it was reported that the phosphatidylinositol 3-kinase signalling system is a master regulator of energy and carbon metabolism [40], regulating the homeostasis of membrane lipids, TAGs, starch, free fatty acids (FFAs) and ATP by influencing other components of the signalling network. Here, we also found that the expression of a carboxypeptidase (B5Y4V6), which negatively regulates membrane lipid metabolism, was upregulated under both LN and HC conditions. The enrichment of upregulated proteins in the phosphatidylinositol signalling system and related genes indicated that this pathway might play important regulatory roles (energy and carbon balance regulation and homeostasis maintenance) in the *P. tricornutum* response to LN conditions. In addition, genes involved in autophagy, plant–pathogen interactions, endocytosis and phagosomes were also enriched, indicating a complex regulatory pattern through signal transduction in *P. tricornutum* under LN conditions.

### Probable role of phenylpropanoids in N recycling under LN and HC conditions

The most upregulated DUP under both LN (790.8-fold change) and HC (287.8-fold change) conditions was PRDX6, which is involved in phenylpropanoid biosynthesis, followed by two predicted transcription factors involved in the regulation of phenylpropanoid biosynthesis ranked second and fifth (LN) and second and fourth (HC) (Tables S2 & S3), indicating an important role for the phenylpropanoid biosynthesis pathway under LN and HC conditions. The phenylpropanoid biosynthesis pathway is involved in the degradation of phenylalanine and tyrosine and thus produces abundant NH_4_^+^, facilitating the recycling of nitrogen under N-limited conditions, while nitrate inhibits large sectors of phenylpropanoid metabolism [41]. The upregulation of the phenylpropanoid biosynthesis pathway might facilitate nitrogen reallocation under both LN and HC conditions.

### Overall response to a high C:N ratio in *P. tricornutum* under HC and LN conditions

**Fig. 9:**
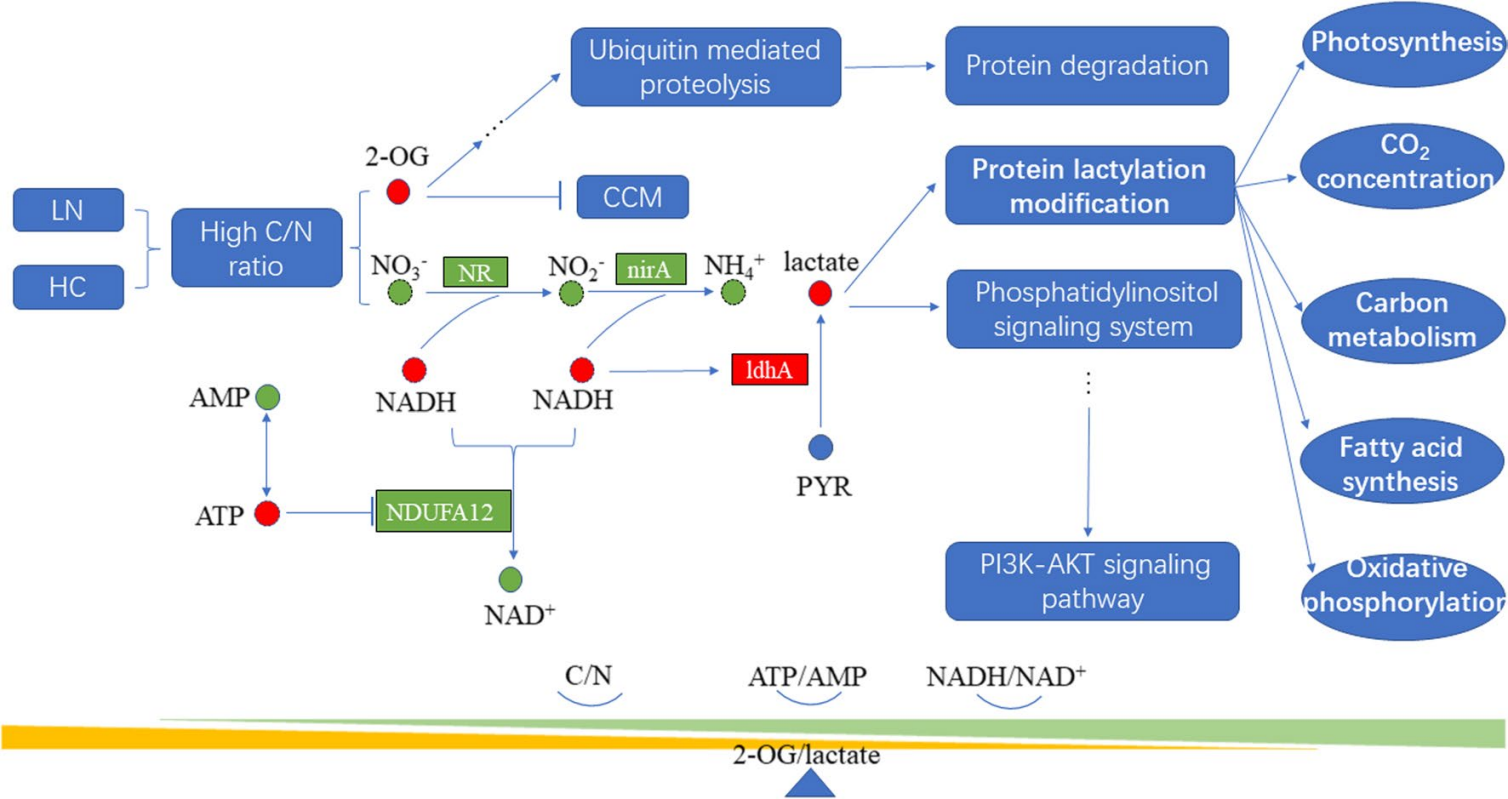
Overall response to high C/N ratio in P. tricornutum under HC and LN conditions. Red, upregulation of metabolites or protein content of genes. Green, downregulation of metabolites or protein contents of genes. Blue, the regulation mood of metabolites or protein content of genes remain unknown. Full line, the regulation of metabolites or proteins are verified by omics data. Dashes lines, the regulation of metabolites or proteins are speculated by related metabolites or proteins. *NDUFA12* NADH dehydrogenase (ubiquinone) 1 alpha subcomplex subunit 12, *NR* nitrate reductase (NAD(P)H), *nirA* ferredoxin-nitrite reductase, *ldhA* d-lactate dehydrogenase. *PYR* pyruvate, *CCM* carbon concentration mechanism

Considering the combined results of the proteome, metabolome and WB analysis and lactylated proteome with respect to *P. tricornutum* under HC and LN conditions, we propose the following responses of *P. tricornutum* to C:N imbalance (Fig. 9): a high C:N ratio stimulates 2-OG accumulation. The resulting nitrogen reduction and nitrite reduction were reduced as a result of N limitation, thereby reducing the consumption of NADH. In addition, accelerated glycolysis, photosynthesis (HC conditions) and the TCA cycle (LN conditions) result in increases in NADH and ATP (Fig. 2). A low AMP:ATP ratio (Table 1) depresses the activity of NADH dehydrogenase, and the accumulation of NADH triggers the activity of *ldh*A (Table 2), resulting in the accumulation of lactate (Table 1). 2-OG and lactate then serve as signalling molecules and regulate a series of pathways. 2-OG senses and transduces signals when the C:N ratio is high, regulates genes involved in the CCM and reduces the extent carbon fixation (Table 2, Fig. 2). Moreover 2-OG can act on the PHD and E3 ubiquitin ligation complex and induce protein degradation through ubiquitin-mediated proteolysis, providing carbon skeletons and NH_4_^+^ for the reallocation of nitrogen. Lactate can regulate the function of proteins through PTM and regulate cellular components through a series of signalling pathways, i.e. IP3K signalling pathways. Lactylated proteins were involved in central carbon metabolism, photosynthesis, oxidative phosphorylation and nitrogen metabolism, thus can maintain C:N balance and energy homeostasis.

## Conclusion

Multiple omics analysis was conducted in *P. tricornutum* under HC and LN conditions. The results indicated 2-OG and lactate was significantly up-regulated under both LN and HC conditions. Genes involved in CCM were down-regulated, indicating 2-OG regulated C:N balance by suppressing CCM-related genes. Genes involved in lactate redundant and energy metabolism are significant regulated. By WB analysis it was proved that lactylation modification occurred in *P. tricornutum*. What is more, the non-histone lactylation modification was enhanced under LN and HC. Lactylated proteomic analysis revealing that lactylation might play an important role in energy and carbon balance regulation and homeostasis maintenance in *P. tricornutum*. Our result reveal C:N regulation mechanism under LN and HC conditions, shedding light on the potential application of *P. tricornutum* to the reduction of CO_2_ emissions from industrial flue gas streams and at the same time acquiring biodiesel raw materials.

## Materials and methods

### Cell culture and treatments

*P. tricornutum* Bohlin was obtained from the Microalgae Culture Center (MACC) at Ocean University of China. Algal cells were cultured using NaHCO_3_-free sterilized artificial seawater supplemented with f/2 [42] under cool white fluorescent light (~ 100 μmol m^–2^ s^–1^) at 20 °C and a 12:12 dark:light cycle. A UV/visible spectrophotometer (UV-1800, Shimadzu, Japan) was used to measure the absorbance at 730 nm (A730 nm) to monitor cell growth. For high CO_2_ (HC, ~ 2000 μatm) or normal CO_2_ (NC, ~ 400 μatm) treatment, the culture was bubbled with ~ 2,000 μatm of CO_2_ or ambient air with a flow rate of 500 ml min^–1^. For the low nitrogen (LN) treatment, a nitrogen-free f/2 medium was produced with artificial seawater and the cultures were bubbled with ambient air at a flow rate of 500 ml min^–1^. For experiments testing the influence of lactate on growth and lipid content, ( ±)-lactate (Xilong Chemical Co., Ltd., Shantou, Guangdong province, China) was added to a final concentration of 1, 5 and 10 mM. Each treatment was administered in triplicate sets. After 8 days, cell pellets were collected and then washed using distilled water at 5,000 g for 4 min. The pellets were frozen in liquid nitrogen and stored at − 80 °C.

### Lipid analysis and metabolomic analysis

Total lipids were extracted and weighed as described by previously [43] with minor modifications. Metabolomic analysis was performed as described [18]. Full details are given in Additional file 1: Methods S1.

### Proteomic analysis

Algal cells were ground into powder in liquid nitrogen, and proteins were extracted using phenol buffer (containing 10 mM dithiothreitol and a 1% protease inhibitor) and lysed by ultrasound. After adding Tris-balanced phenol and centrifuging (4℃, 5500*g*, 10 min), the supernatant was maintained overnight to allow precipitation with ammonium acetate/methanol, and then, it was washed with methanol and acetone. The protein precipitate was dissolved with urea, and the concentration was determined with a BCA protein assay kit (Beyotime). Equal amounts of protein were hydrolysed by trypsin and analysed by LC MS/MS as described in Additional file 1: Methods S1.

The MS/MS raw data were administered by operating MaxQuant (http://www.maxquant.org/) with an integrated Andromeda search engine (v1.6.15.0). Full detail parameters are given in Additional file 1: Methods S1. Significantly differentially expressed proteins were defined by the criteria of a fold change (FC) ≥ 1.5 or ≤ 0.67 (P < 0.05). Functional annotation of the proteins was performed using non-redundant protein (NR), Swiss Protein (SwissProt), Gene Ontology (GO) and KEGG databases.

### Protein validation by parallel reaction monitoring (PRM)

To verify the protein expression levels obtained by label-free analysis, the expression levels of 15 selected proteins, including fructose-bisphosphate aldolase (FbaC5), zeaxanthin epoxidase (ZEP3), beta-glucosidase (bgl), ferredoxin-nitrite reductase (nirA), pyruvate, orthophosphate dikinase (PPdK), nitrate reductase (NR), propionyl-CoA carboxylase (PCCA), d-lactate dehydrogenase (LDHA), glyoxalase (GLO_2 and GLO_1), formidase, precursor of dehydrogenase pyruvate dehydrogenase E1 (PDH1), phosphoenolpyruvate carboxykinase (PEPCK1), ferredoxin-dependent glutamate synthase (GltX) and aconitate hydratase 2 / 2-methylisocitrate dehydratase (acnB), were further quantified by PRM analysis using the original protein samples (NC, LN, and HC) as described in Sect. 2.3. PRM validation was performed as described previously [44]with minor revisions.

### Pan anti-acetylation and pan anti-lactyllysine as determined by Western blotting

Proteins used in Western blotting were extracted according to the same operation manual as used in proteomic analysis except that 3 μM TSA (trichostatin A) and 50 mM nicotinamide were added. Protein concentration was determined by BCA reagent. Western blotting samples were prepared as follows: equal amounts of protein (20 µg/sample) were mixed with SDS-PAGE 4 × loading buffer with a ratio of 3:1 (v/v), heating for 10 min at 95 °C. The same volume of 20% protein marker, boiled samples and 4 × loading buffer were loaded into wells. Gel electrophoresis was then performed as follows: at 80 V for 30 min, then at 120 V until loading dye reach the bottom edge of SDS-PAGE gel. Following transfer the protein to nitrocellulose membrane at 100 V for 1 h at 4 °C, the membranes were blocked in 1 × TBST with 5% non-fat milk with gentle shaking for 1 h at RT. By rinsing with 1 × TBST for three times (10 min each), the membrane was incubated in primary antibody (anti-acetyllysine antibody, PTM-101, Lot: 12838533L303, 1:1000 dilution in 1 × TBST with 2.5% BSA; and pan anti-lactyllysine, PTM-1401RM, Lot: L011121, 1:500 dilution in 1 × TBST with 2.5% BSA) at 4 °C with gentle shaking overnight, rinsing with1 × TBST for 10 min three times, incubating for 1 h at RT with secondary antibody (Thermo, Pierce, goat anti-mouse IgG, H + L, peroxidase-conjugated, 31,430 and 31,460, respectively) diluted 1:10,000 in in 1 × TBST with 5% non-fat milk), rinsing with 1 × TBST for 10 min three times, and staining with ECL western blot detection reagent (Beyotime, Beijing, China) for 2 min, followed by quantification using multiple exposure time to acquire the optimal picture.

### Lactylated proteomic analysis

Protein was extracted and hydrolysed by trypsin as described in 2.3, following by pan-antibody-based PTM enrichment. Briefly, tryptic peptides dissolved in NETN buffer (100 mM NaCl, 1 mM EDTA, 50 mM Tris–HCl, 0.5% NP-40, pH 8.0) were incubated with pre-washed antibody beads (Lot: L011121, PTM Bio) at 4 °C overnight with gentle shaking. After that the beads were washed for four times with NETN buffer and twice with H2O. Then the bound peptides were eluted from the beads with 0.1% trifluoroacetic acid. Finally, the eluted fractions were combined and vacuum-dried and desalted with C18 ZipTips (Millipore) according to the manufacturer’s instructions.

LC MS/MS and database search were conducted as described in 2.3. Full detail parameters are given in Methods S1. For motif analysis, an adjacent sequence model of lactyllysine was analysed by motif-x (http://www.motif-x.med.harvard.edu), integrating 10 amino acids upstream and downstream of the modification sites (lactyl-21-mers). When the number of a lactyl-21-mer is greater than 20 and *P* < 0.000001, the lactyl-21-mer is considered to be a motif of modified peptides. Protein sequences for all *P. tricornutum* proteins were utilized as background databases.

### Statistical analysis

The significance of differences between samples was assessed by two-tailed student *t*-tests and one-way analysis of variance. The statistical significance level was set at *P* < 0.05.

## Supplementary Information

**Additional file 1: Methods S1.** Detail parameters for materials and methods.

**Additional file 2: Table S1.** Annotation combines of proteomics data.

**Additional file 3: Table S2.** Differentially expressed proteins under LN.

**Additional file 4: Table S3.** Differentially expressed proteins under HC.

**Additional file 5: Table S4.** Annotation combines of lactylated proteomics data.

**Additional file 6: Fig. S1.** Overview of proteomics data. (a) Peptide length distribution. (b) Peptide number distribution. (c) Protein coverage distribution. (d) Protein molecular weight distribution.

## Data Availability

The datasets used and/or analysed during the current study are available from the corresponding author on reasonable request.
