# Supplementary material for: Metabolomic, proteomic and lactylated proteomic analyses indicate lactate plays important roles in maintaining energy and C:N homeostasis in Phaeodactylum tricornutum
Source: Biotechnol Biofuels Bioprod. 2022 May 31;15:61. doi: 10.1186/s13068-022-02152-8 (PMC9153171; doi:10.1186/s13068-022-02152-8)
Supplement: Supplementary file 1 — Additional file 1: Methods S1. Detail parameters for materials and methods. [file 13068_2022_2152_MOESM1_ESM.docx]

**Lipid analysis**

Briefly, 20 mg freeze drying cell powder was mixed with 1 mL of chloroform/methanol (1:1) and stirred vigorously for 5 min, then 0.3 mL of 0.2 M H_3_PO_4_ (containing 1 M KCl) was added and mixed. By centrifugation at 5000 × g for 5 min, the solvent phase was recovered and the water phase was discharged. The cell pellets were repeated extracted three times and the solvent phases were combined, evaporated and dried at room temperature under a ventilated fume hood. The total lipid content was obtained by weighing and expressed as percent cell dry weight (% CDW).

**Metabolomic analysis**

Briefly, frozen algal cells were resuspended in a 50:50 (%) methanol:water solution and then centrifuged at 13000 g for 5 min. The supernatants were lyophilized, and the pellets were resuspended in distilled water. DSS (2,2-dimethyl-2-silapentane-5-sulfonate) standard solution (Anachro, Canada) was added and mixed by vortexing. Then, the samples were transferred to 5-mm NMR tubes (Norell, USA), and spectra were collected using a Bruker AV III 600 MHz spectrometer. We used 100-ms mixing and 990-ms pre-saturation times (~80 Hz gammaB1), and the spectra were collected after a total of 128 scans were obtained in a 15-min period at 25°C. Fourier transformation was performed with the free induction decay (FID) signals in a Chenomx NMR Suite, version 8.3 (Chenomx Inc., Edmonton, Canada), and the baseline was corrected. DSS was used as the internal standard, and all the spectra were referenced and analysed against the Chenomx compound library.

**Details for proteomic analysis**

Equal amounts of protein were hydrolysed by trypsin. Trichloroacetic acid (TCA) was added to the samples to a final concentration of 20%, eddy mixed and precipitated at 4 ℃ for 2 h. After centrifugation at 4 500 g for 5 min at 4 ℃, the precipitate was washed with pre-cooled acetone for twice. After the precipitation was dried, a final concentration of 200 mM of tetraethylammonium bromide (TEAB) was added, dispersed by ultrasound, and trypsin was added at a ratio of 1:50 (protease: protein, m/m) for overnight enzymatic hydrolysis. DTT was added to a final concentration of 5 mM and reduced at 56 ℃ for 30 min. Iodoacetamide (IAA) was added to make the final concentration 11 mM and incubated at room temperature for 15 min in dark.

The peptides were dissolved with mobile phase A and then separated with a nanoElute ultrahigh-performance liquid system (phase A consisted of 0.1% formic acid, 2% acetonitrile and 97.9 water, and phase B consisted of 0.1% formic acid and 99.9% acetonitrile). The liquid gradient setting was 6%~24% phase B for 0-70 min followed by 24%~35% phase B (70-84 min), 35%~80% B (84-87 min) and 80% B (87-90 min) at a flow rate of 450 nL min^-1^. The separated peptides were ionized by the capillary ion source and then analysed with a timsTOF Pro mass spectrometer. The voltage of the ion source was 1.7 kV, and the peptide precursor ions and their secondary fragments were detected and analysed by high-resolution TOF MS. The scanning range of the secondary mass spectrometer was 400-1500 m z^-1^.

The MS/MS raw data were administered by operating MaxQuant (http://www.maxquant.org/) with an integrated Andromeda search engine (v1.6.15.0). *P. tricornutom* database was obtained from NCBI and contained 10465 sequences. A reverse database was added to calculate the false positive rate (FDR) caused by random matches, and a common contamination library was added to the database to eliminate the influence of contaminating proteins in the identification results. The parameters are set as follows: Restriction enzyme digestion method: Trypsin/P, Number of missing cleavage site: 2, Minimum peptide length: 7 AA residues; Maximum number of peptide modifications: 5. The mass error tolerance of the primary precursor ion of First search and Main search is set to 20 ppm and 20 ppm, respectively, and the mass error tolerance of the secondary fragment ion is 20 ppm. Carbamidomethylation particular for cysteine, was set up for fix modification, and the variable modification is the oxidation of methionine, the acetylation of the N-terminus of the protein, and the lactation of lysine. The FDR for protein identification and PSM identification is set to 1%. The Maxquant database search results showed the label-free quantification (LFQ) intensity of each protein in different samples (the original intensity value of the protein is corrected between samples). After centralizing the LFQ intensity (*I*) of the protein in different samples, relative quantitative value (*R*) of the protein in different samples was obtained as the follow formula: *R_ij_*=*I_ij_*/*Mean*(*I_j_*) (*i* represents the sample and *j* represents the protein). For multiple replicate sample experiments, calculate the average value of the relative quantitative value of each protein in multiple replicates, and then calculate the ratio of the average of the two samples, and this ratio is used as the final differential expression of the protein between the two samples ratio. Student *t*-test was conducted on the triplicates in each group.

**Details for** **lactylated proteomic analysis**

The MS/MS raw data were administered by operating MaxQuant (http://www.maxquant.org/) with an integrated Andromeda search engine (version 1.5.2.8). The *P. tricornutom* database was obtained from NCBI and contained 10465 sequences. A reverse database was added to calculate the false positive rate (FDR) caused by random matches, and a common contamination library was added to the database to eliminate the influence of contaminating proteins in the identification results. The parameters are set as follows: Restriction enzyme digestion method: Trypsin/P, Number of missing cleavage site: 4, Minimum peptide length: 7 AA residues; Maximum number of peptide modifications: 5. The mass error tolerance of the primary precursor ion of First search and Main search is set to 20 ppm and 20 ppm, respectively, and the mass error tolerance of the secondary fragment ion is 20 ppm. Carbamidomethylation particular for cysteine, was set up for fix modification, and the variable modification is the oxidation of methionine, the acetylation of the N-terminus of the protein, and the lactation of lysine. The FDR for protein identification and PSM identification is set to 1%.
