# Supplementary figures and images for: Metabolomic, proteomic and lactylated proteomic analyses indicate lactate plays important roles in maintaining energy and C:N homeostasis in Phaeodactylum tricornutum
Source: Biotechnol Biofuels Bioprod. 2022 May 31;15:61. doi: 10.1186/s13068-022-02152-8 (PMC9153171; doi:10.1186/s13068-022-02152-8)

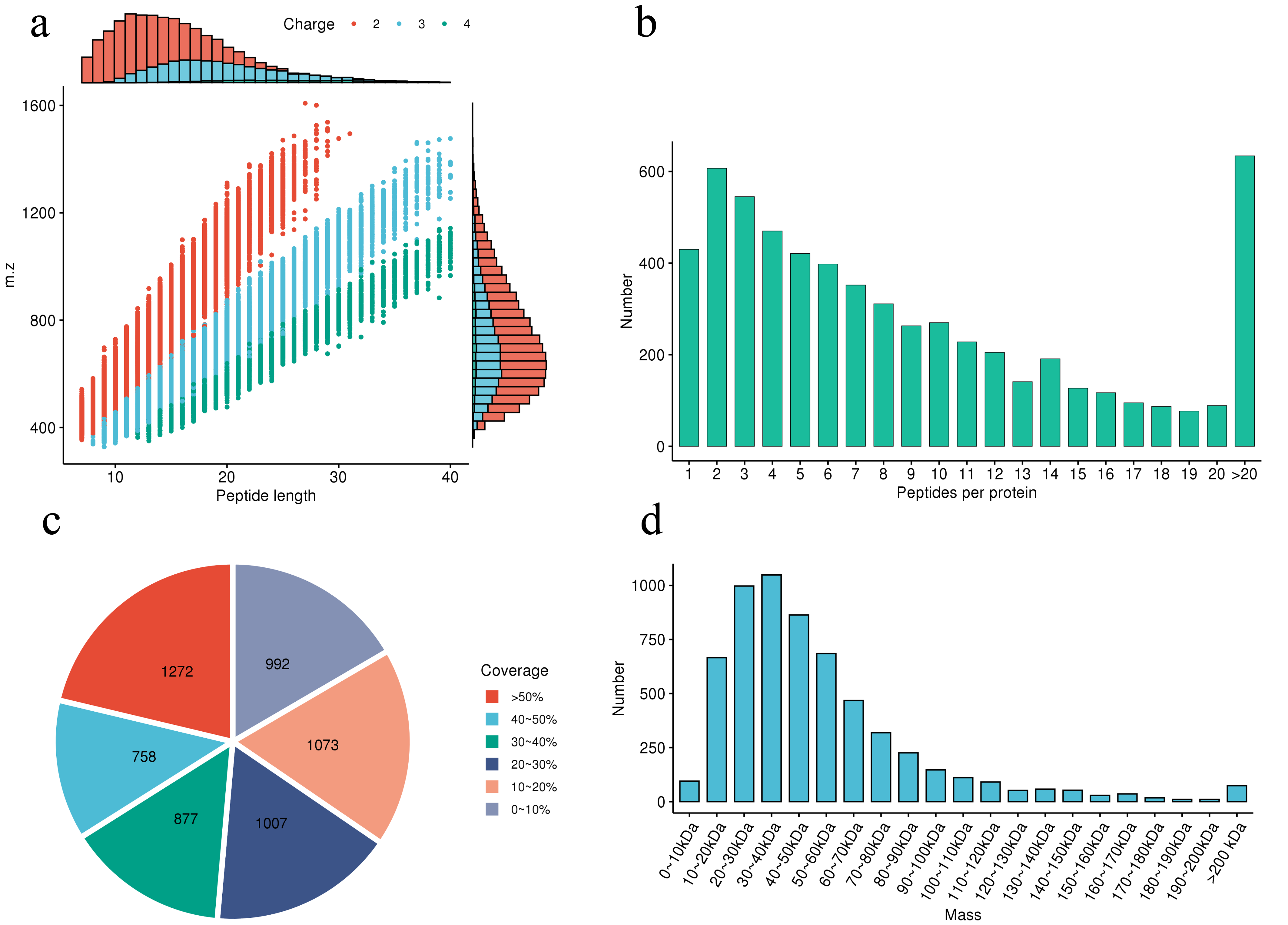

Supplement: Supplementary file 6 — Additional file 6: Fig. S1. Overview of proteomics data. (a) Peptide length distribution. (b) Peptide number distribution. (c) Protein coverage distribution. (d) Protein molecular weight distribution. [file 13068_2022_2152_MOESM6_ESM.tif]
